# Interpersonal Communication Tendency as a Mediator Between Academic Distress and Depression Among Medical Students: A Cross-Sectional Study

**DOI:** 10.31083/AP44018

**Published:** 2025-06-19

**Authors:** Ziyang Zhang, Qingzhi Wang

**Affiliations:** ^1^St. Luke’s College of Nursing, Trinity University of Asia, 1102 Quezon City, Philippines; ^2^Department of Social Medicine and Child Health, School of Public Health, Xuzhou Medical University, 221004 Xuzhou, Jiangsu, China; ^3^Center for Medical Statistics and Data Analysis, Xuzhou Medical University, 221004 Xuzhou, Jiangsu, China

**Keywords:** academic distress, depression, medical students, interpersonal communication, mediator

## Abstract

**Background:**

Medical students frequently encounter high academic demands that increase their risk of depression. Understanding how academic distress interacts with interpersonal communication tendencies to influence depression can inform mental health interventions. This study aims to explore the direct effect of academic distress on depression and the mediating role of interpersonal communication tendency.

**Methods:**

A cross-sectional survey was conducted among 7317 students in medicine and related fields at Xuzhou Medical University from September to December 2022, using stratified random cluster sampling. Data were collected using validated scales measuring academic distress, interpersonal communication tendency, and depressive symptoms. Structural equation modeling was employed to analyze the total, direct, and indirect effects of academic distress on depression, and a multi-group analysis was conducted to examine gender differences.

**Results:**

Academic distress was found to directly and positively affect depression (β = 0.622, 95% CI: 0.556, 0.686). Additionally, interpersonal communication tendency significantly mediated the relationship between academic distress and depression (β = –0.253, 95% CI: –0.294, –0.217). Gender-based analysis indicated a slightly stronger direct effect of academic distress on depression among male students (β = 0.630) compared to female students (β = 0.606).

**Conclusions:**

Academic distress is strongly associated with depression among medical students, with interpersonal communication tendency serving as an effective mediator that reduces depressive symptoms. These findings highlight the need for academic institutions to support medical students by enhancing interpersonal communication skills and providing mental health resources to alleviate academic distress.

## Main Points

1. Academic distress had a significant positive effect on depression among medical students.

2. Interpersonal communication tendency had a significant negative effect on depression among medical students.

3. Interpersonal communication tendency played a mediating role between academic distress and depression.

## 1. Introduction

Depression is a prevalent psychological disorder that poses a significant threat to the mental health of college students in contemporary society [1, 2]. Amid China’s four-decade economic boom, which has been accompanied by significant improvements in living standards and a substantial rise in household income, the educational landscape has undergone notable transformation. The expansion of higher education opportunities, coupled with intensified academic and vocational competition driven by advancements in productivity, have contributed to an escalating trend of psychological and mental health disorders among college students [3, 4, 5]. This trend not only compromises their well-being but also threatens the stability of individuals, families, and society at large. Within this context, depression has emerged as a pivotal health concern, exacerbating both physical and psychological burdens on college students and constituting a leading contributor to the disease burden among the youth demographic [6]. Medical students, in particular, are at an elevated risk of depression, with reported prevalence rates significantly exceeding those of the general college student population [7]. Studies indicate that up to 30% of medical students experience depression, compared to approximately 20% among non-medical college students [3, 8]. This heightened vulnerability may stem from the distinctive challenges inherent in medical education, including prolonged study hours, high levels of academic pressure, exposure to emotionally taxing clinical environments, and uncertainties regarding future career prospects [7]. These factors underscore the importance of focusing research efforts on depression within this specific population.

Students in medicine and related fields (hereinafter collectively referred to as “medical students”), frequently encounter academic distress as they navigate their demanding and extensive curricula [9, 10]. These difficulties are defined as impediments and discomforts within the educational context, arising from an interplay of stemming personal, familial, institutional, and societal factors [11]. Such factors can lead to an inability to focus, diminished academic achievement, and inferior educational outcomes. Among medical students and related fields, these challenges are often compounded by unique stressors, such as the intensive coursework, clinical practice obligations, and societal expectations for professional excellence [12, 13]. These pressures can lead to a higher incidence of mental health challenges among this group [14]. Academic distress has been identified as a significant contributor to depressive symptoms among students [2, 15, 16]. While learning disabilities exert a more profound influence on learning processes, academic distress still poses a notable challenge. It is posited that extended exposure to such negative factors may heighten the risk of depression in college students [17]. The current dearth of empirical evidence supporting a direct link between academic distress and depression among medical students underscores the urgent need for further investigative research in this area.

Coping strategies employed by individuals experiencing academic distress can be broadly categorized as either positive or negative. Research suggests that positive interpersonal communication tendency can mitigate depressive emotions and facilitate adaptation to college life [18]. Positive interpersonal communication tendency refers to the deliberate and non-judgmental focus on specific objects or individuals [19]. Those exhibiting a positive interpersonal communication tendency are more likely to demonstrate superior decision-making abilities, positive thought patterns, and recover more swiftly and effectively from depressive emotions [20]. Furthermore, they can significantly reduce anxiety and depressive emotions triggered by an excessive focus on negative events. Theoretically, this study is grounded in the Stress-Buffering Model [21, 22], which posits that interpersonal factors can mitigate the adverse effects of stress on mental health. This framework suggests that interpersonal communication tendency may play a key role in alleviating the impact of academic distress on depression.

Medical students, equipped with foundational understanding of medical knowledge, are partially capable of addressing the academic distress they encounter and recognize the correlation between academic distress, interpersonal communication tendency, and depression [9, 23]. However, the precise extent and direction of these interrelations remain poorly understood. Specifically, academic distress may influence interpersonal communication tendency by diminishing individuals’ willingness or ability to engage positively in social interactions, as stress often impairs communication behaviors [16, 24]. Conversely, interpersonal communication tendency can influence depression, as positive communication behaviors are associated with improved emotional regulation and reduced depressive symptoms [18]. Therefore, it is hypothesized that interpersonal communication tendency may act as a mediator, buffering the negative effects of academic distress on depression. Based on the above theoretical insights and prior research, this study proposes the following hypotheses: (H1) Academic distress is positively associated with depression; (H2) Interpersonal communication tendency is negatively associated with depression; and (H3) Interpersonal communication tendency mediates the relationship between academic distress and depression.

This study aims to construct a structural equation model to analyze the relationship between academic distress and depressive emotions and to investigate the mediating role of interpersonal communication tendency in this relationship. Meanwhile, there may be gender differences in the relationship between the three main variables, and we will compare the differences by constructing a multicluster model without using gender path coefficients. The objective is to provide empirical evidence and practical insights for the prevention and intervention of depression among medical students, thereby better safeguarding and promoting their mental health.

## 2. Materials and Methods

### 2.1 Participants

This research was conducted within the framework of cross-sectional study. In this study, “medical students” refers to individuals enrolled at Xuzhou Medical University between the years 2018 to 2021, who are pursuing academic programs related to the field of medicine. These students are categorized into various academic specializations, primarily within the disciplines of medicine (such as Clinical Medicine, Dentistry, Public Health, Pharmacy, and Nursing), humanities and social sciences (including Health Management, Medical Psychology, and Hospital Administration), and science and technology (such as Medical Informatics, Medical Artificial Intelligence, Biomedical Sciences, Medical Imaging Engineering, and Biomedical Engineering). The study participants span all four academic years (1st to 4th year), with a sample size of 7317 valid responses collected from a stratified random cluster sampling technique. The survey was conducted over the period from September to December in 2022, with a sample size of 7816 students. Following the exclusion of invalid responses due to missing critical data or outliers, a total of 7317 valid questionnaires were retrieved. This resulted in a response rate of 93.42%. The sample size was determined based on recommendations from the literature, which suggest a minimum of 10–30 participants per observed variable for structural equation model [25]. The total number of observation items for the three core variables in this study is 37, and the required sample size is 370–1100 participants, so the sample size of this study meets the requirements. Ethical approval for the study was granted by the Ethics Committee of Xuzhou Medical University (approval No. XYFY2022-KL157-01). Informed consent was obtained from all participants prior to their inclusion in the study.

### 2.2 Data Collection and Measures

#### 2.2.1 Demographic Characteristics

Basic demographic attributes encompass a spectrum of personal factors, including, but not limited to, age, gender, academic year, hometown, and major.

#### 2.2.2 Interpersonal Communication Tendency Self-Report Scale

Based on previous research [26] and incorporating expert consultation, as well as thorough discussions within the research team, a self-assessment scale for interpersonal communication tendencies was developed, consisting of 6 items (see **Supplementary Table 1**). Each item is rated on a 5-point Likert scale, ranging from 1 (strongly disagree) to 5 (strongly agree). The average score of these items serves as an indicator of interpersonal communication tendencies, with higher scores reflecting a greater inclination toward close interpersonal interactions. In this study, the scale demonstrated strong reliability, with an internal consistency coefficient of Cronbach’s α calculated at 0.895.

#### 2.2.3 The Perception of Academic Stress Scale

The Perception of Academic Stress Scale (PAS) is a widely used instrument for measuring college students’ perceptions of academic stress [27] (see **Supplementary Table 2**). The PAS comprises three subscales: (1) Stress related to academic expectations (4 items), (2) Stress related to faculty work and examinations (8 items), and (3) Stress related to students’ academic self-perception (6 items). Participants rate each item on a 5-point Likert scale, ranging from 1 (strongly disagree) to 5 (strongly agree). The overall PAS score is calculated by summing the item scores and dividing by the total number of items (18), yielding an average score that serves as an indicator of overall academic stress. Higher scores reflect greater levels of academic distress. Items 9, 13, 14, and 15 are scored in reverse. In this study, the internal consistency of the scale, as measured by Cronbach’s α, was 0.904, indicating high reliability.

#### 2.2.4 Depression Subscale of Symptom Checklist-90

The Depression subscale of Symptom Checklist-90 (SCL-90) was used to assess the depression status of medical students [28] (see **Supplementary Table 3**). Each item was rated on a 5-point scale (0 = Not at all, 1 = A little bit, 2 = Moderately, 3 = Quite a bit, 4 = Extremely), with a total of 13 items used to measure depression. The average score across all items is used as the indicator of depression severity. The internal consistency coefficient of the scale for this study is Cronbach’s α = 0.931.

### 2.3 Statistical Analysis

Data entry was conducted using Epidata3.10 software (The EpiData Association, Odense, Denmark), with double-entry and consistency checks to ensure data accuracy and reliability. For statistical analyses, SPSS 26.0 (IBM Corp., Chicago, IL, USA) was used for reliability analysis, descriptive statistics, and correlation analysis. Continuous variables (including age, interpersonal communication tendency, academic distress, and depression) were described using means and standard deviations (SDs), while categorical variables (including gender, academic year, hometown, and major) were summarized with frequency analysis. Reliability analysis was performed using Cronbach’s alpha (α) coefficient, with coefficients exceeding 0.7 deemed acceptable [29]. Pearson correlation analysis was used to assess the relationships between academic distress, interpersonal communication tendency and depression.

Based on the hypothesized model aligned with the study’s objectives, Amos 24.0 (IBM Corp., Chicago, IL, USA) software was employed to construct and fit the initial model. Parameter estimation for the model was carried out using Maximum Likelihood Estimation (MLE), with path coefficients representing the strengths of relationships between variables, and standardized path coefficients (β) used for interpretation. The Bootstrap method was applied to test the mediation effect model. Model fit indices were assessed across three categories: (1) Absolute Fit Indices: Root Mean Square Residual (RMR) <0.05, Root Mean Square Error of Approximation (RMSEA) <0.08, and both the Goodness-of-Fit Index (GFI) and Adjusted Goodness-of-Fit Index (AGFI) >0.90. (2) Incremental Fit Indices: Normed Fit Index (NFI), Relative Fit Index (RFI), Tucker-Lewis Index (TLI), and Comparative Fit Index (CFI), each >0.90. (3) Parsimonious Fit Indices: Chi-Square to Degrees of Freedom ratio (χ^2^/df) <5, with the Parsimony Goodness-of-Fit Index (PGFI) >0.50 [30]. During model refinement, non-significant paths were removed, and model fit indices were re-evaluated. If fit remained suboptimal, modification indices (MI) were used to identify and establish covariances between residuals, with each modification followed by re-running the analysis until the fit indices fell within acceptable thresholds. Finally, we constructed a multicluster model to validate the differences in path coefficients between genders. Statistical significance was set at α = 0.05.

## 3. Results

### 3.1 Demographic Characteristics

The demographic characteristics of the study participants are presented in Table 1. A total of 7317 college students participated, completing all required assessments, with a mean age of 21.62 years and 41.00% identifying as male. Participants were relatively evenly distributed across academic years (1st to 4th year), with academic specializations primarily in medicine (63.58%), humanities and social sciences (18.50%), and science and technology (17.92%). Additionally, the majority of participants (60.13%) reported rural origins.

**Table 1.** **Demographic characteristics (N = 7317)**.

Characteristic		N/Mean	%/SD
Age		21.62	2.37
Gender			
	Male	3000	41.00%
	Female	4317	59.00%
Academic year			
	1st year	1958	26.75
	2nd year	1816	24.83
	3rd year	1888	25.80
	4th year	1655	22.62
Hometown			
	Urban area	2917	39.87
	Rural area	4400	60.13
Major ^a^			
	Humanities & Social Sciences	1354	18.50
	Medicine and health	4652	63.58
	Science and Technology	1311	17.92

Note: ^a^ Humanities & Social Sciences include disciplines such as Health Management, Medical Psychology, and Hospital Administration; Medicine and health encompass fields such as Clinical Medicine, Dentistry, Public Health, Pharmacy, and Nursing; Science and Technology cover areas including Medical Informatics, Medical Artificial Intelligence, Biomedical Sciences, Medical Imaging Engineering, and Biomedical Engineering.

### 3.2 Correlation Analysis 

The correlation between interpersonal communication tendency, academic distress, and depression are shown in Table 2. The average score of interpersonal relationship tendency, academic distress, and depression was 1.37 ± 0.93, 1.72 ± 0.45 and 1.49 ± 0.43, respectively. Academic distress is positively correlated with both interpersonal communication tendency (r = 0.618, *p* < 0.001) and depression (r = 0.622, *p* < 0.001). Interpersonal communication tendency is negatively correlated with depression (r = –0.409, *p* < 0.001).

**Table 2.** **Correlation between interpersonal communication tendency, academic distress, and depression among medical students**.

Item	Mean ± SD	1	2	3
1 Interpersonal communication tendency	1.37 ± 0.93	1		
2 Academic distress	1.72 ± 0.45	0.618***	1	
3 Depression	1.49 ± 0.43	–0.409***	0.622***	1

Note: ***p < 0.001.

### 3.3 Mediating Effect Model of Interpersonal Communication Tendency Between Academic Distress and Depression

**Fig. 1.**
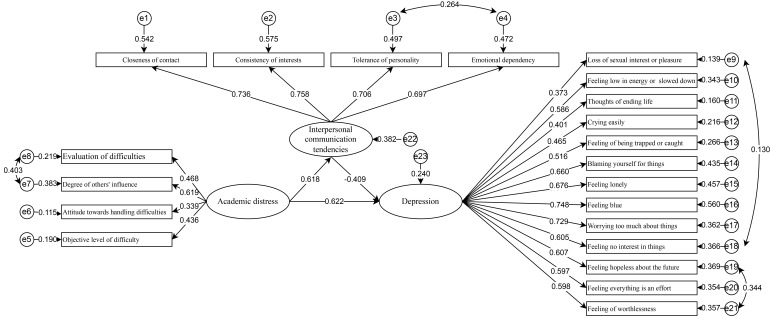
**The mediating effect model of interpersonal communication tendency between academic distress and depression among medical students**.

A structural equation model was used to construct the mediating effect model of interpersonal communication tendency between academic distress and depression. The initial model results indicated that χ^2^/df, RFI, and TLI did not meet the model fit criteria. Consequently, modifications were made based on the modification indices (MI), allowing covariances to be established between residuals. Following each modification, the analysis was re-run until the fit indices reached acceptable thresholds. After modification, the χ^2^/df was greater than 5 and *p* < 0.001. However, when the sample size is very large (such as in this study with N = 7317), χ^2^/df is not a reliable fit index. It is necessary to ensure that NFI, RFI, TLI, CFI, GFI, and AGFI are all greater than 0.90, and that RMSEA and RMR are both less than 0.08. Thus, the fit indices of the modified model met the required standards, as shown in Table 3. The path diagram of the modified model is illustrated in Fig. 1.

**Table 3.** **Fit indexes for the mediation effect model of interpersonal communication tendency on the relationship between academic distress and depression among medical students**.

Model	χ²/df	NFI	RFI	TLI	CFI	GFI	AGFI	RMSEA	RMR
Initial model	23.702	0.904	0.892	0.896	0.908	0.944	0.931	0.056	0.026
Revised model	5.466	0.963	0.957	0.961	0.967	0.978	0.972	0.034	0.021
Multicluster model	5.874	0.948	0.947	0.955	0.957	0.969	0.965	0.026	0.030

Note: χ^2^/df, ratio of chi-square value to degrees of freedom; NFI, Normed Fit Index; RFI, Relative Fit Index; TLI, Tucker-Lewis Index; CFI, comparative fit index; GFI, goodness-of-fit index; AGFI, adjusted goodness-of-fit index; RMSEA, root mean square error of approximation; RMR, root mean square residual.

Using Bootstrap testing with 2000 resamples, the structural equation model analysis showed the total effect, direct effect, and indirect effect for the full model, as well as for male and female groups. The results are presented in Table 4. In the full model, academic distress has a direct effect on depression (β = 0.622, 95% CI: 0.556, 0.686). Additionally, academic distress has an indirect effect on depression through interpersonal communication tendency (β = –0.253, 95% CI: –0.294, –0.217). In the multi-group analysis, similar results were found, for the male group, academic distress has a direct effect on depression (β = 0.630, 95% CI: 0.547, 0.717), and an indirect effect on depression through interpersonal communication tendency (β = –0.254, 95% CI: –0.304, –0.212). For the female group, academic distress has a direct effect on depression (β = 0.606, 95% CI: 0.531, 0.690), and an indirect effect on depression through interpersonal communication tendency (β = –0.244, 95% CI: –0.293, –0.204).

**Table 4.** **The mediating effect of interpersonal communication tendency between academic distress and depression among medical students**.

Effect relationship		Pathway	Effect size	Boot SE	95% CI
Full Model					
	Total Effect	Academic distress → Depression	0.369	0.022	(0.325, 0.410)
	Direct Effect	Academic distress → Depression	0.622	0.034	(0.556, 0.686)
	Indirect Effect	Academic distress → Interpersonal communication tendency → Depression	–0.253	0.020	(–0.294, –0.217)
Male group					
	Total Effect	Academic distress → Depression	0.377	0.028	(0.322, 0.430)
	Direct Effect	Academic distress → Depression	0.630	0.044	(0.547, 0.717)
	Indirect Effect	Academic distress → Interpersonal communication tendency → Depression	–0.254	0.023	(–0.304, –0.212)
Female group					
	Total Effect	Academic distress → Depression	0.362	0.026	(0.313, 0.415)
	Direct Effect	Academic distress → Depression	0.606	0.041	(0.531, 0.690)
	Indirect Effect	Academic distress → Interpersonal communication tendency → Depression	–0.244	0.022	(–0.293, –0.204)

“→” represents the directional relationship of influence in the pathway.

## 4. Discussion

To our knowledge, this study is the first to examine the mediating role of interpersonal communication tendencies in the relationship between academic distress and depression among students in medicine and related fields (hereinafter collectively referred to as “medical students”). Our findings indicate that a stronger tendency for interpersonal communication can reduce levels of depression in medical students and that interpersonal communication serves as a significant mediator between academic distress and depression.

### 4.1 The Direct Positive Effect of Academic Distress on the Depression 

Our study showed that academic distress had a direct positive effect on depression among medical students, which supported Hypothesis 1. This is consistent with the previous findings [2, 31]. Academic distress refers to the psychological strain arising from academic demands, including excessive workloads, strict deadlines, and the constant pressure to excel [32]. Medical students are particularly susceptible to these stressors due to the distinctive nature of their education, which encompasses demanding coursework, rigorous examinations, and intensive clinical training [33]. The cognitive stress theory of depression suggests that negative cognitive patterns, when combined with chronic stressors such as academic distress, can significantly increase the risk of depression [34]. This interaction creates a vicious cycle: students’ difficulties in managing academic stress contribute to emotional exhaustion, which further impairs their capacity to cope, thereby intensifying depressive symptoms [8]. In China, the strong societal emphasis on academic success underscores its role as a primary pathway to upward social mobility [35]. This cultural value is further amplified by China’s competitive higher education system, the largest in the world, which produces over 10 million graduates entering an increasingly saturated labor market annually [36]. The scarcity of postgraduate opportunities has resulted in intense competition, driving many students to prepare for the national graduate entrance exam [37]. For Chinese medical students, the pressure is compounded by the need to excel academically to obtain scholarships, adapt to challenging academic environments, and meet rigorous graduation requirements, such as thesis defenses [38]. From a stress-response perspective, exposure to prolonged academic stress triggers negative evaluations in the cerebral cortex, leading to depressive emotional states [2]. Stressors deplete psychological reserves and impair students’ ability to engage in effective coping strategies, fostering a state of helplessness and exacerbating depressive symptoms. For medical students, the intensity and persistence of academic stressors increase the likelihood of entering this maladaptive cycle.

### 4.2 Interpersonal Communication Tendency is Negatively Associated With Depression

Our study showed that interpersonal communication tendency had a negative effect on depression among medical students, which supported Hypothesis 2. This aligns with prior research indicating that positive psychosocial resources, including social support and interpersonal communication, play a vital role in alleviating depressive symptoms and fostering mental health [39]. Interpersonal communication acts as a multidimensional resource, influencing psychological well-being through physiological, emotional, and social mechanisms. Individuals with stronger interpersonal communication skills exhibit enhanced resilience, as they are better equipped to navigate stressors and maintain stable mental health outcomes under adversity [40]. This resilience stems from their ability to mobilize social resources, gain reassurance, and receive constructive feedback, which strengthens their capacity to cope. From a physiological perspective, effective interpersonal communication reduces stress-induced neurohormonal fluctuations, such as excessive cortisol secretion, and helps regulate emotional responses, thereby mitigating the biological impact of stress [18, 41]. Interpersonal communication also functions as a key coping strategy by enabling individuals to express emotions, seek advice, and share burdens, thereby reducing feelings of isolation and helplessness [42]. For medical students, whose demanding academic environments often lead to stress and burnout, these interactions provide critical emotional support and validation. Engaging in meaningful conversations fosters a sense of belonging, counters feelings of alienation, and creates a supportive environment that guards against psychological distress [43].

### 4.3 The Mediating Role of Interpersonal Communication Tendency

Interpersonal communication tendency mediates the association between academic distress and depression, providing valuable insights into how social relationships can mediate the relationship between academic stress and mental health outcomes. This supports Hypothesis 3. The mediation is evident in two key aspects: the positive correlation between academic distress and interpersonal communication, and the negative association between interpersonal communication and depression. When faced with academic stress, students often adopt adaptive coping strategies by seeking social support, which helps them share concerns, seek advice, and gain emotional reassurance [44]. This proactive engagement not only enhances their interpersonal interactions but also fosters collaborative learning and problem-solving, particularly in the challenging context of medical education [45]. Meanwhile, higher interpersonal communication tendencies mitigate depressive symptoms by serving as a psychological buffer. The buffering model posits that social support protects individuals from the detrimental effects of stress by fulfilling the need for belonging, fostering positive emotional states, and dispersing worries [45, 46]. Empirical studies further confirm that interpersonal communication acts as a buffer against the adverse effects of stressful life events, promoting psychological well-being [47, 48]. This protective mechanism operates through enhanced emotional regulation strategies, such as cognitive reappraisal, enabling individuals to effectively manage negative emotions and maintain mental health [49]. Moreover, individuals with strong interpersonal communication networks leverage resources like understanding, encouragement, and support from peers and family to sustain emotional stability and resilience, thereby reducing the impact of academic distress on depression [50].

### 4.4 Gender Differences in the Direct and Indirect Effects of Academic Distress on Depression

The results indicate a slight difference in the direct and indirect effects of academic distress on depression across male and female students. Specifically, while the direct impact of academic distress on depression is slightly stronger in males than in females, the indirect effects through interpersonal communication tendency are comparable but again marginally higher for males. This variation may suggest that males may experience a more immediate impact of academic distress on depressive symptoms, possibly due to differences in coping styles or societal expectations surrounding the expression of academic struggles [51, 52]. In contrast, females might engage in slightly different patterns of communication, which may help buffer some of the impact of academic stress on depression [53]. These findings highlight the importance of developing gender-sensitive interventions in academic settings, particularly those that address communication skills and emotional resilience to better support students facing academic pressure.

### 4.5 Implications for Practice

The findings of this study have important implications for practice, particularly in addressing mental health challenges among medical students. First, the direct impact of academic distress on depression underscores the need for academic institutions to implement stress-reduction strategies tailored to medical education. Universities should provide resources such as time management training, workload adjustments, and regular mental health screenings to identify at-risk students early. Second, the protective role of interpersonal communication tendency highlights the importance of fostering social support systems within academic environments. Institutions can encourage peer-to-peer interaction by creating mentorship programs, group-based learning activities, and support networks that facilitate open communication and emotional sharing. Faculty and staff should also receive training on how to cultivate a supportive academic atmosphere that normalizes seeking help and prioritizes mental well-being. Third, the mediating role of interpersonal communication tendency suggests that interventions promoting communication skills can have a dual benefit: reducing depression while helping students manage academic stress. Workshops on effective interpersonal communication, emotional expression, and active listening should be integrated into the curriculum. Additionally, counseling services should emphasize the role of social engagement and provide opportunities for group therapy or peer-led support sessions. Overall, a comprehensive approach combining academic policy reforms, targeted communication training, and gender-sensitive mental health interventions can help create a more supportive environment for medical students, ultimately enhancing their psychological resilience and academic performance.

### 4.6 Strengths and Limitations

A key strength of this study is its comprehensive analysis of how academic distress relates to interpersonal communication tendency, and depression among medical students. To the best of our knowledge, this is the first study to explore the role of interpersonal relationships in the relationship between academic distress and depression among medical students. The novelty of this study lies in its identification of interpersonal communication as a crucial mediator, emphasizing how communication tendencies can mitigate the adverse effects of academic distress on depression. This provides new insight into the complex interplay between academic stress and mental health in medical students, offering a fresh perspective on potential interventions. However, there are some limitations to this study: First, the sample was restricted to medical students at Xuzhou Medical University, which may limit the generalizability of findings to broader student populations or to students in different academic and cultural settings. Future studies should consider diverse cohorts to enhance representativeness. Second, the cross-sectional design limits causal inferences between academic distress, interpersonal communication tendency, and depression. Longitudinal studies would be valuable in establishing causality and tracking changes over time. Third, self-reported data were used to measure interpersonal communication tendencies, academic stress, and depression, which could introduce bias due to social desirability or inaccuracies in self-assessment. Integrating objective measures or external assessments may provide a more comprehensive view. Additionally, while rigorous statistical modeling techniques were employed, potential model modification during data analysis could raise concerns about overfitting. Replication in future studies is advised to confirm the model’s robustness and stability.

## 5. Conclusions

This study provides a comprehensive analysis of the relationships between academic distress, interpersonal communication tendency, and depression among medical students. The findings affirm that academic distress significantly increases depressive symptoms and that interpersonal communication tendency serves as a key mediating factor. Students with stronger communication tendencies report lower depression levels, suggesting that effective communication serves as a protective mechanism against academic stress. These findings underscore the importance of mental health resources and interventions in medical schools, particularly programs that enhance interpersonal communication skills and address gender-specific needs. By fostering a supportive academic environment, institutions can help students better manage academic pressure and safeguard their mental well-being.

## Data Availability

The original contributions presented in the study are included in the article, further inquiries can be directed to the corresponding author.

## Supplementary Material

Supplementary material associated with this article can be found, in the online version, at https://doi.org/10.31083/AP44018.
