# Supplementary material for: Interpersonal Communication Tendency as a Mediator Between Academic Distress and Depression Among Medical Students: A Cross-Sectional Study
Source: Alpha Psychiatry. 2025 Jun 19;26(3):44018. doi: 10.31083/AP44018 (PMC12231427; doi:10.31083/AP44018)
Supplement: Supplementary file 1 [file 2757-8038-26-3-44018-s1.docx]

Supplementary

Items from this standardized questionnaire are used to obtain students' agreement on interpersonal communication tendencies according to Campbell et al.

Table S1 Interpersonal preference Tendency Self-Assessment Scale

| Items | Options |
| --- | --- |
| 1. People tell me that I am easy to talk to. | 1=Strongly disagree;  2=Disagree;  3=Neutral;  4=Agree;  5=Strongly agree |
| 2. Strangers often approach and start talking to me. |  |
| 3. People tell me I am a good listener. |  |
| 4. I am honest with others about my thoughts and feelings. |  |
| 5. I believe that communication will be productive. |  |
| 6. I use examples to help me explain what I am talking about. |  |

Table S2 Academic distress Self-Report Scale

| Items | Options |
| --- | --- |
| **Stresses related to academic expectations** | 1=Strongly disagree;  2=Disagree;  3=Neutral;  4=Agree;  5=Strongly agree |
| 1. Competition with my peers for grades is quite intense |  |
| 2. My teachers are critical of my academic performance |  |
| 3. Teachers have unrealistic expectations of me |  |
| 4. The unrealistic expectations of my parents stress me out |  |
| **Stresses related to faculty work and examinations** |  |
| 5. The time allocated to classes and academic work is enough |  |
| 6. The size of the curriculum (workload) is excessive |  |
| 7. I believe that the amount of work assignment is too much |  |
| 8. Am unable to catch up if getting behind my work |  |
| 9. I have enough time to relax after work (-) |  |
| 10. The examination questions are usually difficult |  |
| 11. Examination time is short to complete the answers |  |
| 12. Examination times are very stressful to me |  |
| **Stresses related to students’ academic self-perceptions** |  |
| 13. Am confident that I will be a successful student (-) |  |
| 14. Am confident that I will be successful in my future career (-) |  |
| 15. I can make academic decisions easily (-) |  |
| 16. I fear failing courses this year |  |
| 17. I think that my worry about examinations is weakness of character |  |
| 18. Even if I pass my exams, am worried about getting a job |  |

Note：Items 9,13,14, and 15 are reverse scores.

Table S3 Depression subscale of symptom checklist 90 Scale

| Items | Options |
| --- | --- |
| 1. Loss of sexual interest or pleasure | 0=Not at all  1=A little bit  2=Moderately  3=Quite a bit  4=Extremely |
| 2. Feeling low in energy or slowed down |  |
| 3. Crying easily |  |
| 4. Feeling of being trapped or caught |  |
| 5. Blaming yourself for things |  |
| 6. Feeling lonely |  |
| 7. Feeling blue |  |
| 8. Worrying too much about things |  |
| 9. Feeling no interest in things |  |
| 10. Feeling hopeless about the future |  |
| 11. Feeling everything is an effort |  |
| 12. Feelings of worthlessness |  |
| 13. Thoughts of ending your life |  |
